# Book Reviews

**Published:** 1875-11

**Authors:** 


### Book Reviews.

[NOTE. — All works reviewed in the pages of the CHICAGO MEDICAL JOURNAL AND EXAMINER may be found in the extensive stock of W. B. KEEN, COOKE & Co., whose catalogue of Medical Books will be sent to any address upon request.]

THE MUCOUS MEMBRANE OF THE UTERUS, WITH SPECIAL REFERENCE TO THE DEVELOPMENT AND STRUCTURE OF THE DECIDUÆ. By *Geo. J. Engelmann, A.M., M.D.* With fourteen illustrations. 8 vo., 65 pages. (Reprinted from the *American Journal of Obstetrics.*) New York: William Wood & Co. 1875.

The great advances in medical science which have characterized the past twenty-five years, may be very largely ascribed to the patient investigations of microscopists. The labors of Virchow, Billroth, Klob, Rindfleisch and others, have completely revolutionized the views formerly held, respecting many important subjects in the domains of physiology and pathology. The *brochure* before us, occupies a place in the same field of work, and although it inculcates, as we believe, some erroneous views in regard to certain subjects—notably in the matter of the relations existing between menstruation and ovulation—it is nevertheless a valuable addition to the literature of a deeply interesting and important theme.

The work is the result of a series of microscopic investigations, pursued during the fall of 1871 by the author, in connection with Dr. Kundrat, first assistant to Prof. Rokitansky. The scene of these researches was, chiefly, the pathological laboratory of the General Hospital of Vienna, than which no place, perhaps, could have afforded better facilities for studies of this character. In addition, much material was afforded by the postmortems of the Charité and the obstetrical clinic of Berlin during the summer of 1872, and also the extensive collection of ova in the anatomical museum of the latter city.

During the course of these labors, over three hundred uteri were examined, and the condition of the uterine mucous membrane noted in all its various changes from the time of its first appearance in the fœtus, through its period of development to maturity and its season of functional activity, down to the time of involution and inaction.

An account of these researches was published in Stricker's "Medizinische Jahrbücher" for 1873; but, according to our author, now a resident of St. Louis, the MS. for that publication was prepared after his departure from Vienna, by Dr. Kundrat, and contained some views which he cannot endorse; and this fact, together with some others, has induced him to issue the present work.

The book is divided into three parts, as follows: Part I. The Mucous Membrane of the Womb, in its Development up to the Time of Puberty. Part II. The Mucous Membrane of the Womb during its Period of Maturity and Functional Activity, from the Time of Puberty to the Change of Life. Part III. The Mucous Membrane after the Change of Life.

Under the first head we are informed that in its earliest phases of development, the uterine mucous membrane consists simply of round or polygonal cells imbedded in a net-work of connective tissue. No glandular structure is apparent until about the tenth year, although traces of the presence of glands, in the shape of small crypt-like depressions, are found as early as the third or fourth year. From the tenth year forward, the changes both in the increasing thickness of the membrane and the development of the glands, proceed rapidly to maturity; although, owing to the great differences of race and nationality among the inhabitants of Vienna, and the fact that many of the uteri examined were those of girls whose health had been greatly impaired by chronic and wasting diseases, the author was unable to fix definitely the time of full development.

In Part II, the author first considers the condition of the fully matured membrane during its period of rest.

At this time it is described as a delicate, soft, pale-gray or grayish-red layer, which, owing to the entire absence of a submucous areolar tissue, is closely attached to the muscular structure beneath. Its thickness is 0.04 inch at the fundus and anterior and posterior walls, but thinner at the sides, cervix and tubar ostia. Its glands are destitute of basement membrane, and appear simply as epithelial tubes, imbedded in the substratum of connective tissue.

During the menstrual period the mucous membrane exhibits very different conditions from those just described. "It is swollen, 0.118—0.236 in thickness, of an almost pulpy consistence. Its surface is puffy, wavy, and in places reveals the delicately injected capillaries, after removal of its coating of whitish, opaque mucus, occasionally tinged with blood."

This increased thickness of the membrane is due to hypertrophy of its superficial layers, resulting from a proliferation of round cells and an enlargement of those previously existing, as well as an increase of the intercellular substance. The glands themselves are very much enlarged, especially those portions of them near the surface. The vessels, too, are greatly increased in size and gorged with blood, although there is no new vascular formation.

Dr. Engelmann does not endorse the opinion promulgated by Tyler Smith, Pouchet and others, that the mucous membrane is shed at each menstrual period; and he states that "in not one of the many uteri examined at such periods, was the mucous membrane, or even its superficial layer, found wanting." A few pages further on, however, he uses language, which, if not actually contradictory of the above, at least conveys a different impression. In speaking of the retrograde metamorphosis of the tissue, consequent on the fatty degeneration which accompanies the menstrual process, he says: "The destruction and detachment of a large part of the more exposed elements of the surface, and even of the glandular epithelium, accompanies this process—in proof of which I will say that numerous epithelial cells, in a state of beginning fatty degeneration, are found imbedded in the whitish, somewhat bloody, mucus which fills the uterine cavity."

The latest writer who has touched specially upon this point, is Dr. John Williams. (On the Structure of the Mucous Membrane of the Uterus and its Periodical Changes, *Obstetrical Journal of Great Britain and Ireland*, February and March, 1875.) He states positively that in two of the uteri examined by him, the membrane of the cavity of the uterine body was wholly absent. In one case the woman, aged thirty-five years, died about the ninth day of typhoid fever. The menstrual flow had been present for four days, and had not quite ceased at the time of death.

In the second case, although no menstrual history could be obtained, the appearances were similar. Here, too, the mucous membrane, perfect in the cervix of the organ, ceased abruptly at the internal os. In both cases the muscular wall was exposed in the cavity.

These observations of Dr. Williams confirm those previously made by Tyler Smith, Handfield Jones, Pouchet and others.

In regard to the cause of the menstrual flow, the author has only an opinion to give. During and immediately after the hæmorrhage, a fatty metamorphosis involves the cells of the upper layers of the membrane, the blood vessels, and the glandular and surface epithelia. These changes he regards as the cause of the hæmorrhage. He says: "Thus it appears that after a certain degree of tumefaction has been reached by the menstrual membrane, this change in its elements, this process of disintegration is inaugurated, and rapidly developing, especially upon the surface, leads to the rupture of the distended vessels, and hæmorrhage ensues."

Dr. Engelmann is a believer in the correctness of the ovulation theory of menstruation, and thinks the evidences of the simultaneous occurrence of these processes, are quite conclusive. He states that the specimens examined by him go far toward proving that the rupture of the Graafian follicle generally occurs towards the close of the menstrual period.

In support of this opinion he cites some cases, two of which are remarkable for not having any bearing upon the question whatever. Of them he says, "the mucous membrane already displayed the menstrual tumefaction, and no signs of retrograde metamorphosis; in these uteri menstruation had not yet taken place, but was evidently soon to be expected; and no sign of a recently ruptured follicle was to be found in the ovaries." Menstruation was not present, and there were no signs of ovulation; hence, menstruation depends upon ovulation!

While we have neither the disposition nor the space to enter upon a discussion of this matter, we will merely state here that we regard the evidence that has been adduced in favor of the truth of the ovulation theory, to be far outweighed by that which has been brought to oppose it.

In the joint publication of Drs. Engelmann and Kundrat, a novel theory, originally advanced by Loewenhardt, was endorsed. According to this view, the impregnated ovum belonged, not to the menstruation last preceding conception, but to the period following, when, although the ordinary tumefaction of the membrane took place, no discharge occurred. Dr. E. regards this theory as erroneous and untenable, and contends that it is not supported by the anatomical facts observed by Dr. K. and himself. He urges also that the doctrine would make the duration of pregnancy three-fourths of a lunar month less than is generally accepted, and that it implies rupturing of the Graafian follicle at a period long prior to the menstrual hæmorrhage, conclusions to which he cannot assent.

The theory so long maintained, and still taught, of the origin of the decidua reflexa by the pushing forward of the mucous membrane of the ovum in its passage from the tube into the cavity of the womb, is wholly refuted by the examinations made of menstrual uteri. In every case examined, the openings of the Fallopian tubes, as well as the internal os uteri, were completely pervious, and in no case was there any union or even agglutination of the membrane at these places; and hence, the ovum could not possibly produce a reduplication of the mucosa in the manner formerly supposed.

The author next describes the normal development of the deciduæ, and traces their growth from the first two weeks of conception to the end of pregnancy.

He maintains that the usually accepted notion, that the villosities of the chorion penetrate the mouths and ducts of the uterine glands, is erroneous. He characterizes it as a very plausible theory, but one not founded upon any macroscopic or microscopic preparations in the hands of any of the authors who advocate its truth. He says: "My examinations and still preserved specimens, prove that no such relation exists between the ovum and the uterine membranes, that the villi do not enter the gland tubes, but that the adhesions existing are owing to an agglutination of the parts and to the growth of the serotinal tissue around the villi." He maintains also that this statement with regard to the relation between the chorion and serotina, is equally true of the more fully developed ovum, and that hence, the very foundation of the wide-spread theory of the formation of the placenta, falls to the ground.

There seems to be some difficulty in deciding whether the large blood sinuses of the placental portion of the serotina, which all authors describe as the most important feature of that structure, are normal and necessary, or whether they are artificial and accidental. It is well known that Ercolani and Braxton Hicks deny their existence; the latter, having examined eight placenta *in situ* in different stages of pregnancy, states that he found no blood, or only a mere trace, between the villi.

While the injections of the placenta made by Dr. Engelmann, or under his direction, were conducted in the most careful manner, and the results were analogous to those of other observers, he does not feel sure that the injected fluid may not have ruptured the delicate walls of the capillaries, and thus have produced the puzzling sinuses.

A chapter devoted to the regeneration of the mucous membrane after parturition, is exceedingly interesting. This process begins in some cases as early as the third week after delivery, at which time the inner surface of the uterus is "lined with a very thin, smooth membrane of new formation, which is still covered by more or less of a pale-yellowish mucus, sometimes streaked with blood."

At this time the membrane, although frail and delicate, and not over 0.006 inch in thickness, is perfect in its elements, the glandular structure, interstitial tissue and surface ephithelium being all represented. The restitution of the membrane to its full growth and normal appearance, and the complete involution of the organ, are accomplished by the end of the fifth week, except at the placental site, where the work of regeneration progresses more slowly. Many chronic diseases retard, and in some cases would seem to prevent altogether, the involution and regeneration of the organ.

Dr. Engelmann believes that when, under any circumstances, the entire decidua becomes detached, a perfect mucous membrane can never be reproduced; and that "in those cases, whether of tedious instrumental delivery, or of spontaneous expulsion of the ovum, in which the membrane is *apparently* expelled *in toto*, the lowest meshes with the fundi of the glands, or, in extreme cases, only the exposed fu di, still some part of the mucosa remains *in utero;* the muscular fibres are not laid bare, and restitution is readily explained."

Here again the views of our author come in conflict with those of other observers. Both Kölliker and Heschl maintain that during parturition the mucous membrane of the uterus is entirely removed, and the muscular fibres become exposed in the uterine cavity. The former writer states that "the mucous membrane is completely thrown off after parturition, in the form of decidua and placenta uterina, and this has to be formed entirely new." Heschl says: "The placenta site, which always occupies a third part of the contracted uterus, still retains a projecting, uneven, and considerably lacerated surface. The rest of the inner surface of the body of the uterus, is composed of muscular substance, from which, here and there, hang shreds, the remains of the decidua."

The condition of the mucous membrane after the change of life is dismissed in a single paragraph of ten lines, the substance of which may be summed up in two words —rest and atrophy. A. R. J.

